# Untargeted Metabolomic Plasma Profiling of Emirati Dialysis Patients with Diabetes versus Non-Diabetic: A Pilot Study

**DOI:** 10.3390/biom12070962

**Published:** 2022-07-08

**Authors:** Bayan Hassan Banimfreg, Hussam Alshraideh, Abdulrahim Shamayleh, Adnane Guella, Mohammad Harb Semreen, Mohammad Tahseen Al Bataineh, Nelson C. Soares

**Affiliations:** 1 Department of Industrial Engineering, College of Engineering, American University of Sharjah, Sharjah P.O. Box 26666, United Arab Emirates; g00080088@aus.edu (B.H.B.); halshraideh@aus.edu (H.A.); 2 Nephrology Department, University Hospital Sharjah, Sharjah P.O. Box 72772, United Arab Emirates; adnane.guella@uhs.ae; 3 Department of Medicinal Chemistry, College of Pharmacy, University of Sharjah, Sharjah P.O. Box 27272, United Arab Emirates; msemreen@sharjah.ac.ae (M.H.S.); nsoares@sharjah.ac.ae (N.C.S.); 4 Sharjah Institute for Medical Research, University of Sharjah, Sharjah P.O. Box 27272, United Arab Emirates; 5 College of Medicine, University of Sharjah, Sharjah P.O. Box 27272, United Arab Emirates; 6 College of Medicine and Health Sciences, Department of Genetics and Molecular Biology, Khalifa University of Science and Technology, Abu Dhabi P.O. Box 127788, United Arab Emirates; 7 Center for Biotechnology, Khalifa University of Science and Technology, Abu Dhabi P.O. Box 127788, United Arab Emirates

**Keywords:** diabetic kidney disease, hemodialysis, LC-MS/MS, untargeted metabolomics

## Abstract

Diabetic kidney disease (DKD) is a severe irreversible complication of diabetes mellitus that further disturbs glucose metabolism. Identifying metabolic changes in the blood may provide early insight into DKD pathogenesis. This study aims to determine blood biomarkers differentiating DKD from non-diabetic kidney disease in the Emirati population utilizing the LC-MS/MS platform. Blood samples were collected from hemodialysis subjects with and without diabetes to detect indicators of pathological changes using an untargeted metabolomics approach. Metabolic profiles were analyzed based on clinically confirmed diabetic status and current HbA1c values. Five differentially significant metabolites were identified based on the clinically confirmed diabetic status, including hydroxyprogesterone and 3,4-Dihydroxymandelic acid. Similarly, we identified seven metabolites with apparent differences between Dialysis Diabetic (DD) and Dialysis non-Diabetic (DND) groups, including isovalerylglycine based on HbA1c values. Likewise, the top three metabolic pathways, including Tyrosine metabolism, were identified following the clinically confirmed diabetic status. As a result, nine different metabolites were enriched in the identified metabolic pathways, such as 3,4-Dihydroxymandelic acid. As a result, eleven different metabolites were enriched, including Glycerol. This study provides an insight into blood metabolic changes related to DKD that may lead to more effective management strategies.

## 1. Introduction

Diabetes Mellitus (DM) is a chronic metabolic disorder disease with ever-increasing prevalence among middle eastern populations. The hallmark of diabetes, hyperglycemia, is harmful to many organ systems, primarily the cardiovascular, ophthalmic, and renal systems. These long-term complications substantially worsen quality of life [1]. In 2019, the International Diabetes Foundation (IDF) estimated 4.2 million deaths among adults worldwide due to diabetes and its complications, including chronic kidney disease (CKD) [2]. CKD has become a primary worldwide health concern due to the high mortality rate [3,4]. Individuals with CKD are five to ten times more susceptible to premature death than to progress to end-stage renal disease (ESRD) [5]. Individuals with ESRD will routinely undergo hemodialysis to compensate for the failing kidney function. DM is a leading cause of ESRD [6,7]. Inversely, the renal function progressive decline and CKD-related sequelae also disturb glucose metabolism [8]. This association has been of long-standing interest. Cardiovascular mortality and progression to ESRD are the two significant unmet medical needs in patients with CKD and DM. Diabetic patients undergoing hemodialysis have a lower survival rate than non-diabetic patients with ESRD due to other renal diseases [9,10]. Hemodialysis is a frequent procedure to compensate for the failing kidney function, resulting in a constant shift in the metabolic profile. For example, a recent study found that almost one-third of diabetic hemodialysis patients might face impulsive solutions of hyperglycemia with glycated hemoglobin (HbA1c) levels less than 6% [11]. This uncertain biological plausibility and these unspecified medical consequences are a phenomenon called “Burnt-Out Diabetes” [12]. Furthermore, several glucose-lower agents and their active metabolites are metabolized in the kidneys and emitted, requiring dosage correction or avoidance in hemodialysis patients [12]. Therefore, diabetic nephropathy under routine hemodialysis will encounter hyperglycemia and hypoglycemia via multifactorial processes relating to kidney dysfunction, the uremic environment, and hemodialysis [12,13,14,15,16]. The quest for predictive and surrogate endpoint biomarkers for advanced DKD has received significant interest [7]. Nevertheless, there are no novel biomarkers in the clinical or trial set [17]. Several studies have shown potential biomarkers of DKD. The primary metabolites were products of lipid metabolism (such as esterified and nonesterified fatty acids, carnitines, phospholipids), branch-chain amino acid and aromatic amino acid metabolism, carnitine, and tryptophan metabolism, nucleotide metabolism (purine, pyrimidine), and the tricarboxylic acid cycle or uraemic solutes [17,18,19,20]. Moreover, mitochondrial function and fatty acid oxidation play a crucial part in the DKD progress [21,22]. These studies demonstrated substantial variations in the metabolomic profiles, perhaps due to differences in geography, ethnicity, sample selection, and analytical platform. Little is known about the metabolomic profile of DKD under hemodialysis from the middle eastern populations. Therefore, this study explores the metabolomic profile of diabetic and non-diabetic United Arab Emirates (UAE) citizens (known as Emirati) undergoing hemodialysis to uncover the potential novel biomarkers in this population. However, diabetic medication intake for dialysis patients is expected to have effects on their metabolic profiling. Therefore, we also analyzed the data based on the available HbA1c values. Metabolomics utilizes quantifiable metabolites from specimens to obtain helpful information on the physiological state. Chronic diseases occur from the impact of multiple factors, such as genetics, lifestyle, and environment. Comparing the metabolite concentration levels in phenotypically recognized populations, e.g., diseased and control subjects, might support identifying pathways and biological activities linked with a specific disease. Metabolism refers to the biochemical interactions within an organ system to maintain essential processes. Metabolomics is a rapidly emerging area of translational research with promising abilities to identify biochemical compounds that can serve as the early diagnostic, therapeutic, and prognostic value of chronic diseases such as diabetes mellitus. A metabolomics experiment involves targeted metabolomics and untargeted metabolomics approaches [23]. The untargeted metabolomics approach is the qualitative or semiquantitative analysis of the feasible primary number of metabolites from different biological and chemical classes in a physical specimen [24]. Recently, the investigation of DKD via metabolomics has been of primary interest [21,25]. Despite the increased interest in metabolomics in DKD patients [26,27], more studies need to be conducted in such a manner. Specifically, studies on diabetics under hemodialysis have been rare. This study explores the blood metabolic profile for UAE Dialysis patients with diabetes and without diabetes.

## 2. Materials and Methods

### 2.1. Ethics Statement

Hospital Ethics and Research Committee, a local research ethics committee at the University Hospital Sharjah, UAE, approved the study protocol (REF number: UHS-HERC-012-10062019). All volunteers were supplied with an information sheet explaining study objectives, design, and confidentiality. Written informed consent was obtained from all participants of the study.

### 2.2. Study Design

We conducted a single-site cross-sectional study, and all available patients were recruited. However, the sample size is constrained by the available resources, such as individuals’ willingness to participate and the cost of sample analysis. Therefore, 36 subjects from Emirati citizens who were treated at University Hospital Sharjah were selected.

### 2.3. Sample Collection and Preparation

A measure of 4 mL of blood was then collected from each subject into a sterile container. The fresh blood samples were spun down in a centrifuge and the extracted plasma was collected and frozen at −80 °C for long-term storage until further metabolomics analysis. The blood was never frozen as it would make it difficult to recover the plasma. All samples were collected daily (between 8 and 10 am) pre-dialysis while fasting. An aliquot of plasma sample was placed into a microcentrifuge tube and cold methanol was added into the sample at 3:1 *v/v* (i.e., 30 µL sample, add 90 µL cold methanol). The mixture was vortexed and sat at −20 °C for two hrs. Next, the samples were centrifuged at 20,817× *g* for 15 min at 4 °C. Then, the supernatant was transferred to a new microcentrifuge tube. Three times the original sample volume was transferred (i.e., for 30 µL sample, add 90 µL cold methanol, then transfer 90 µL supernatant). The sample was dried using a Speed vac at 30–40 °C. The dried sample was stored in a −80 °C freezer for further use or dissolved in solvent for LCMS analysis. Dissolved samples are preferably in the starting solvent (0.1% formic acid) where volume is three times the original plasma volume. For example, when 30 µL serum/plasma has been used, the supernatant is dissolved in 90 µL 0.1% formic acid. The vials were placed in the autosampler.

### 2.4. Analytical Analysis: Liquid Chromatography-Mass Spectrometry (LC-MS/MS)

TimsTOF Mass Spectrometer (BRUKER, Karlsruhe, Germany) and Metaboscape software version 4 were employed to separate and detect the cell metabolites. It was equipped with a trapped quadrupole time-of-flight mass spectrometer and comprised a Solvent delivery systems pump (ELUTE UHPLC Pump HPG 1300), Autosampler (ELUTE UHPLC), Thermostat column compartment (ELUTE UHPLC), Computer System, Windows 10 Enterprise 2016 LTSB, Data Management Software, Bruker Compass HyStar 5.0 SR1 Patch1 (5.0.37.1), Compass 3.1 for otofSeries, and otofControl Version 6.0. Metabolites were analyzed in auto MS/MS positive scan mode within the range of 20–1300 *m*/*z* utilizing electrospray ionization (ESI). The ESI source was 10 L/min, and the drying temperature was equal to 220 °C. The capillary voltage of the ESI was 4500 V with 2.2 bar nebulizer pressure. The collision energy was set at 7 eV and end Plate Offset as 500 V. A HAMILTON^®^ Intensity Solo 2 C_18_ column (100 µm × 2.1 mm × 1.8 µm) was utilized for the separation of metabolites, and Sodium Formate was used as a calibrant for the external calibration step. Solvent A (Water +0.1% FA) and solvent B (Acetonitrile + 0.1% FA) were used in gradient elution mode for metabolite analysis. Metabolites were analyzed in auto MS/MS positive scan mode within the range of 20–1300 *m*/*z* utilizing electrospray ionization (ESI). The ESI source with dry nitrogen gas was 10 L/min and the drying temperature equal to 220 °C. The capillary voltage of the ESI was 4500 V with 2.2 bar nebulizer pressure. For MS^2^ acquisition, the collision energy was set at 20 eV and end Plate Offset as 500 V. A Hamilton^®^ Intensity Solo 2 C_18_ column (100 mm × 2.1 mm × 1.8 µm) was utilized for the separation of metabolites, and sodium formate was used as a calibrant for external calibration step. For metabolite analysis, solvent A (Water + 0.1% FA) and solvent B (Acetonitrile + 0.1% FA) were used in gradient elution mode. The gradient program used a flow rate of 0.250 mL/min with 99A:1.0B from 0.00–2.00 min, 99A:1.0B to 1.0A:99B from 2.00–17.00 min, 1.0A:99B from 17.00–20.00 min, 1.0A:99B to 99A:1.0B from 20.00–20.10 min, flow rate of 0.350 mL/min with 99A:1.0B from 20.10–28.50 min, flow rate of 0.250 mL/min, with 99A:1.0B from 28.50–30 min giving a total run time of 30 min with a maximum pressure of 14,993 pounds per square inch (PSI). The autosampler temperature was set at 8 °C and the column oven temperature at 35 °C. A total volume of 10 µL was injected into the QTOF MS. The flow rate was set as (0.250–0.350 mL/min) for 30 min in gradient mode with a maximum pressure of 14,993 psi. The elute autosampler temperature was set at 8 °C, and the column oven temperature was set to 35 °C. A total volume of 10 µL was injected into the QTOF MS. LC total ion chromatograms (TIC) and fragmentation patterns of the metabolites were identified by MetaboScape^®^ version 4.0 (Bruker-Daltonics, Billerica, MA, USA) and MS/MS library search based on the Bruker HMDB Metabolite Library 2.0 (Bruker Daltonics, Billerica, MA, USA). The latter library provides more than 6000 MS/MS spectra for more than 800 compounds selected from the Human Metabolome Database (HMDB) [28]. Data processing: Processing and statistical analyses were performed using MetaboScape^®^ 4.0 software (Bruker Daltonics, Billerica, MA, USA). Bucketing in T-ReX 2D/3D workflow, the parameters set for molecular feature detection were as follows: minimum intensity threshold equal to 1000 counts along with minimum peak length of 7 spectra for peak detection, using peak area for feature quantitation. The mass recalibration was performed within a retention time range between 0–0.3 min. Only those features present in at least 3 of 12 samples (per cell type) were considered. On the other hand, the MS/MS import method was set to be performed by average. The parameters for data bucketing were assigned as follows: Retention time range started at 0.3 min and ended at 25 min, while mass range started at 50 *m*/*z* and ended at 1000 *m*/*z*. Each sample was run in duplicate LC-MS/MS analysis as described above.

### 2.5. Statistical Analysis

R software version 4.0.5 was used for the statistical analysis [29]. Data were analyzed in a duplicate technique. Data were cleaned to exclude which concentration values were missing or below the detection limit. The average for each sample was obtained. Data were standardized, and normalization was performed through Logarithmic transformation. Metabolic profiles were analyzed based on (1) clinically confirmed diabetic status and (2) current HbA1c values to account for the disease control status.

Differential metabolites between the 11 DD and 25 DND patients were detected using Principal Component Analysis (PCA) and Wilcoxon rank-sum test (known as Mann–Whitney U-test). In addition, the False Discovery Rate (FDR) method was applied to adjust for the multiple comparisons problem. The criteria of differential metabolite determination are as follows: *q* value (FDR adjusted *p*-value) < 0.05. In all statistical tests, a significance level of 0.05 was used.

The metabolomics experiment usually results in a high number of significant metabolites. Therefore, we focused on the over-represented subsets in the outcomes to ease interpretation. Pathway analysis is to reduce dimensionality and simplify functional understanding. The Pathway Analysis module for pathway enrichment and topological analysis in the MetaboAnalyst platform [30] was utilized for identified pathways analysis.

## 3. Results

### 3.1. Clinical Data of Patients

We enrolled 36 subjects who were being treated at University Hospital Sharjah, UAE. There were 20 females aged between 56 and 85 (average: 69.9 ± 8.16 years; median: 69 years), and 16 males aged between 34 and 90 (average: 73.68 ± 13.07 years; median: 74 years). Out of the 36 participants, 11 were hemodialysis diabetic patients (6 females, 5 males), and 25 were non-diabetic hemodialysis patients (14 females, 11 males). The classification for patients is based on the clinically confirmed diabetic status according to WHO diagnostic criteria for diabetes (fasting plasma glucose ≥7.0 mmol/L (126 mg/dL) or 2 h. plasma glucose ≥11.1 mmol/L (200 mg/dL)). However, we will classify the patients based on their most recent HbA1c values for further analysis. The patients were elderly with renal complications of diabetes. Our data show that most known diabetic hemodialysis patients have their diabetes controlled (72.3%). Surprisingly, about 32.0% of the known non-diabetic hemodialysis patients did not control their blood glucose. There is no statistically significant difference in age, gender, HbA1c, cholesterol total, and blood Hb between DD and DND groups (*p* > 0.05).

### 3.2. Differential Metabolite Screening

**Figure 1:**
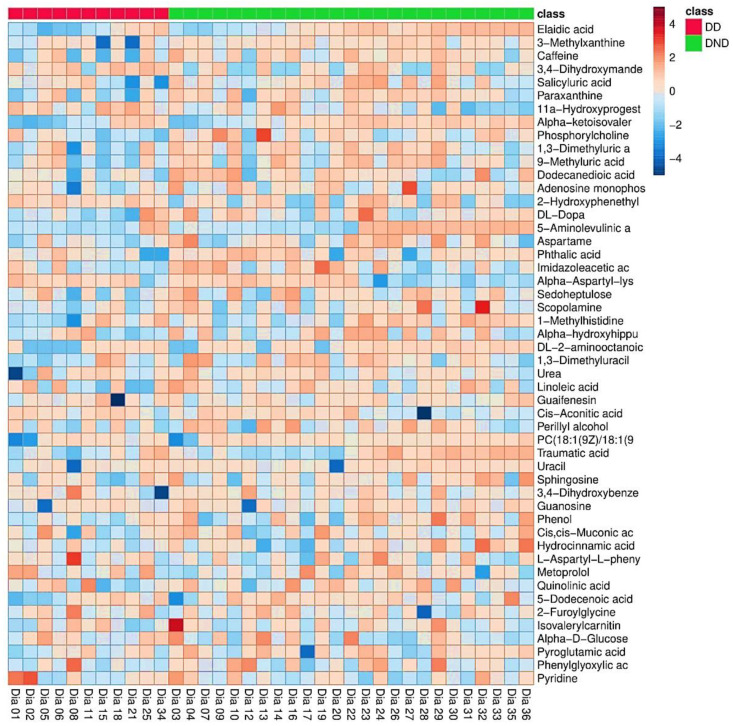
Heatmap of the 50 selected metabolites among the DD and DND patients (clinically confirmed diabetic status). The columns represent samples, the rows represent metabolites, and the relative content of the metabolites is displayed by color. The heatmap shows detected metabolites among DD and DND groups.

Using the LC-MS-MS technique and HMDB database [28], 142 metabolites were detected and identified. These detected and identified metabolites were documented. In addition, we used the MetaboAnalyst platform to examine the patterns of features. The top 50 metabolites based on the differences in averages between DD and DND groups are displayed as a heatmap in Figure 1. Heatmap in Figure 1 shows detected metabolites among DD and DND groups. The color gradient demonstrates concentration levels for each metabolite in each sample. Heatmap in Figure 1 indicates no apparent differences in the concentration of the metabolites among the two groups. Potential differences exist between some metabolites by initial visualization inspection, such as Alpha-Aspartyl-Lys and Cis-Aconitic Acid (Figure 1). However, robust and advanced statistical tools should test these “potential” metabolites.

Heatmaps of the complete list of metabolites shown based on the clinically confirmed diabetic status and HbA1c values grouping scenarios are provided as Figure S1.

### 3.3. Multivariate Statistical Analysis

**Figure 2:**
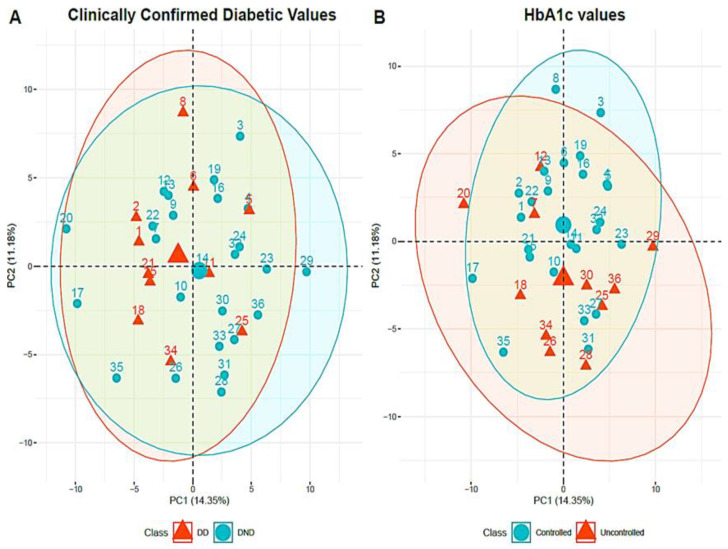
Plots of PCA scores: (**A**) PCA plot based on clinically confirmed diabetic status; (**B**) PCA plot based on latest HbA1c values (controlled if HbA1c value is less than 6.5% and uncontrolled otherwise).

As stated previously, statistical analysis was performed based on (1) clinically confirmed diabetic status, (2) HbA1c values. Plots of the top two principal components (PCs) following the PCA analysis of the 142 identified metabolites under the two scenarios considered are shown in Figure 2. Figure 2A shows a PCA plot following known diabetic status for patients grouping. The plot in Figure 2A depicts that the blood components of the DD group and DND group did not have apparent clustering indicating almost similar metabolic profiles among the two groups. Therefore, the latest available HbA1c values for both groups were used for further PCA analysis. Figure 2B shows the PCA plot following participants’ grouping based on their latest HbA1c value (controlled if HbA1c value is less than 6.4% and uncontrolled otherwise). Figure 2B illustrates an improved separation among the controlled and uncontrolled groups. Participants with uncontrolled blood glucose tend to have lower values of PC2 compared to the participants with controlled blood glucose.

### 3.4. Discrepancy Metabolite Analysis

**Figure 3:**
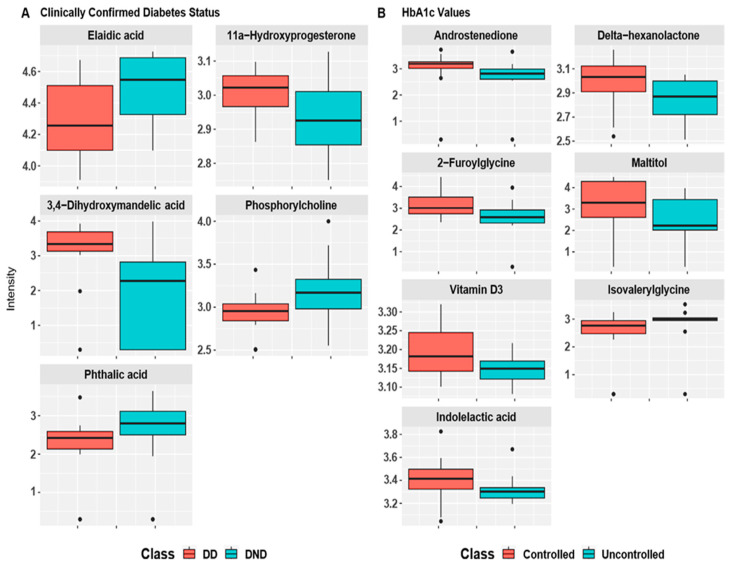
(**A**) Boxplot of normalized intensity metabolites for the clinically confirmed diabetic status; (**B**) boxplot of normalized intensity metabolites based on latest HbA1c values.

Wilcoxon rank-sum test as a robust non-parametric testing procedure was used to examine the differential metabolites among participants groups under the two analysis scenarios as discussed previously. First, we conducted the Wilcoxon rank-sum test for all 142 detected metabolites using clinically confirmed diabetic status. Then, FDR adjusted *p*-values were obtained. Out of the 142 metabolites, five metabolites significantly had different concentrations among the DD and DND groups. Boxplot of these metabolites intensities are shown in Figure 3A with adjusted *p*-values of Elaidic acid (*p* = 0.036), Phosphorylcholine (*p* = 0.036), and Phthalic acid (*p* = 0.036), the levels of 11a-Hydroxyprogesterone (*p* = 0.036), and 3,4-Dihydroxymandelic acid (*p* = 0.036).

Analysis was repeated according to the latest HbA1c values as controlled or uncontrolled. Boxplots of the identified significant metabolites according to the HbA1c values are shown in Figure 3B. These boxplots show significant difference in the levels of Androstenedione (*p* = 0.042), Delta-hexanolactone (*p* = 0.042), 2-Furoylglycine (*p* = 0.042), Maltitol (*p* = 0.045), Vitamin D3 (*p* = 0.045), and Indolelactic acid (*p* = 0.049) and the levels of Isovalerylglycine (*p* = 0.049).

**Figure 4:**
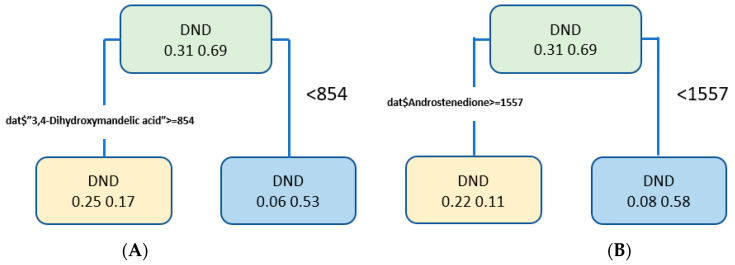
Decision tree plots: (**A**) Decision tree plot for the clinically confirmed diabetes data; (**B**) decision tree plot for the HbA1c data.

We have applied an rpart decision tree to evaluate the results in both cases. The performance measures for the clinical confirmed diabetes data are 77% accuracy, 60% sensitivity, and 90% specificity. However, the HbA1c data performance measures are 81% accuracy, 67% sensitivity, and 88% specificity. Figure 4 shows the tree plot for two cases.

### 3.5. Analysis of Metabolic Pathways

**Figure 5:**
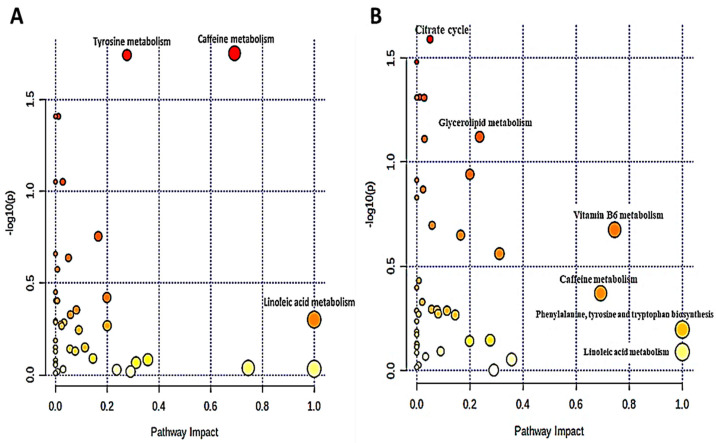
(**A**) Metabolic pathway analysis of clinically confirmed diabetic status; (**B**) metabolic pathway analysis based on latest HbA1c values. Each bubble in the bubble diagram represents a metabolic pathway. Color gradient and circle size indicate the significance of the pathway ranked by *p*-value (yellow: higher *p*-values and red: lower *p*-values) and pathway impact score (the larger the circle, the higher the pathway impact score). The top three metabolic pathways were identified by name according to the -log(*p*) value and pathway impact score.

To understand the metabolic pathways involved in the development of diabetes, we used the MetaboAnalyst platform to perform pathway enrichment and topological analysis of differential metabolites in blood [30]. First, we examined the pathway analysis for the clinically confirmed diabetic status. Based on the identified metabolites in our data, the metaboAnalyst platform detected 46 metabolic pathways, as exhibited in Figure 5A. According to the -log(*p*) value and pathway impact score, the top three metabolic pathways were selected, which are: Tyrosine metabolism, Linoleic acid metabolism, and Caffeine metabolism. Metabolic pathway analysis results show nine different metabolites enriched in these three metabolic pathways: Linoleic acid, Glycerophosphocholine, Paraxanthine, Caffeine, 3,4-Dihydroxymandelic acid, 3,4-Dihydroxyphenylglycol, 3,4-Dihydroxybenzeneacetic acid, DL-Dopa, and L-Tyrosine, as shown in Table 1. A Wilcoxon rank-sum test was used to analyze the differential metabolites enriched in the identified pathways. The levels of Tyrosine metabolism-related metabolite 3,4-Dihydroxymandelic acid (*p* = 0.028) are noticeably different in both groups. However, there was no significant difference in the levels of other metabolites between the DD group and the DND group.

The same approach was applied considering the grouping of participants based on the latest HbA1c values. In this case, 46 metabolic pathways were screened by the MetaboAnalyst platform. Figure 5B shows the top six selected metabolic pathways based on -log(*p*) value and pathway impact score, which are: Citrate cycle, Glycerolipid metabolism, Vitamin B6 metabolism, Caffeine metabolism, Phenylalanine, tyrosine, tryptophan biosynthesis, and Linoleic acid metabolism. Metabolic pathway analysis results indicated 11 different metabolites enriched in these six metabolic pathways: Cis-Aconitic acid, Glycerol, Pyridoxal 5′-phosphate, Pyridoxal, 4-Pyridoxic acid, Caffeine, Paraxanthine, L-Phenylalanine, L-Tyrosine, Linoleic acid, and Glycerophosphocholine, as shown in Table 1. A Wilcoxon rank-sum test was used to analyze the differential metabolites enriched in the identified metabolism pathways. The levels of glycerolipid metabolism-related metabolite Glycerol (*p* = 0.050) were significantly different among the controlled and uncontrolled groups. However, there was no significant difference in the levels of other metabolites between the two groups.

## 4. Discussion

This study used LC-MS/MS to examine blood metabolites of DD and DND Emirati patients. LC-MS/MS provides a state-of-the-art quantitative determination of biological compounds with high specificity, sensitivity, and throughput [31]. The analysis is two-fold: (1) the analysis based on clinically confirmed diabetic status; (2) the analysis based on the available HbA1c values. We detected and identified 142 metabolites among the DD and DND groups. Initial results using PCA of clinically confirmed diabetic status showed that DD and DND of the plasma components could not have apparent clustering. Therefore, we further performed PCA using HbA1c values. The results showed that the uncontrolled group could be clearly distinguished from the controlled group, indicating that the controlled and uncontrolled groups’ plasma metabolites are different. Subsequently, the Wilcoxon rank-sum test and metabolic pathway analysis of 142 metabolites were performed. Differential metabolites analysis based on the clinically confirmed diabetic status between both groups showed enrichment of Hydroxyprogesterone (*p* = 0.036) and was consistent with previous publications related to androgenic metabolism, oxidative stress, and adipocyte accumulation among the DD group [32,33]. Therefore, the inability to metabolize androstenedione to testosterone and accumulation in blood among the DD group could be correlated with DKD, and thereby, a useful biomarker. Moreover, we detected an alteration in norepinephrine derivative, 3,4-Dihydroxymandelic acid (*p* = 0.036) turnover and metabolism among the DD group and consistent another diabetic sequela such as diabetic cardiomyopathy [34]. Similarly, we identified higher levels of isovalerylglycine (*p* = 0.049) among the uncontrolled group based on HbA1c values. Interestingly and consistent with a previous study that concluded a higher clearance rate among DKD compared to vascular causes of kidney disease [35]. Vitamin D is an essential regulator of calcium and phosphate homeostasis. Surprisingly, despite the expected decline in kidney function, including 1α-hydroxylation (a necessary step in vitamin D metabolism), we detected an increase in vitamin D3 (*p* = 0.045) among the controlled group based on HbA1c values [36]. Perhaps due to compensatory mechanisms by other organs such as the gastrointestinal system, a previous study showed a protective role against creatinine degradation among individuals with diabetes with high HbA1c values [37]. Furthermore, plasma metabolites of the Glycerolipid metabolism pathways such as Glycerol (*p* = 0.05) were increased in the uncontrolled group based on HbA1c values. Interestingly, a previous study concluded that altered tissue lipid metabolism is involved in the pathogenesis of toxin-induced nephropathy and can be used as an early screening biomarker [38].

Last, mitochondrial dysfunction is one of the mechanisms contributing to the incidence and development of DKD [39,40,41]. Mitochondrial dysfunction is associated with kidney disease in non-diabetic contexts, and increasing evidence indicates that dysfunctional renal mitochondria are pathological mediators of DKD [40]. In addition, studies revealed that fatty acid metabolism disorders contribute to developing DKD in T2DM patients [42]. For example, previous western studies concluded that the lower intake of polyunsaturated fatty acids, primarily linolenic and linoleic acid, is associated with CKD in T2DM patients [43,44]. Our study found that the DD group decreased elaidic acid (*p* = 0.036). Therefore, targeting key enzymes for such metabolites may be a promising avenue in treating DKD, especially advanced-stage DKD such as ESRD.

We acknowledge the limitation of the small number of patients enrolled in this study. In addition, this one-site pilot study requires a follow-up with a larger cohort to validate our findings further. Furthermore, some of the identified metabolites, such as caffeine, can be further attributed to other factors such as diet and medication-the need for further validation.

In conclusion, metabolomics is an emerging technology with an essential role in understanding health and disease conditions as metabolic biomarkers have translational potential to improve disease diagnosis and therapeutic targets. Herein, we identified for the first-time potential biomarkers, such as isovalerylglycine, elaidic acid, hydroxyprogesterone, 3,4-Dihydroxymandelic acid, and glycerolipid metabolites such as Glycerol for early detection of DKD based on robust metabolomics modeling between diabetic hemodialysis and non-diabetic hemodialysis patients in the UAE population.

## Figures and Tables

**Table 1:** Analysis of the top metabolic pathways based on clinically confirmed diabetic status and latest HbA1c values.

	Name	-Log(*p*)	Impact	Compounds	Pathway
Clinically confirmed diabetic status	Linoleic acid metabolism	0.30064	1.0	Linoleic acid, Glycerophosphocholine	hsa00591
	Caffeine metabolism	1.7512	0.69231	Paraxanthine, Caffeine	map00232
	Tyrosine metabolism	1.7414	0.27636	3,4-Dihydroxymandelic acid, 3,4-Dihydroxyphenylglycol, 3,4-Dihydroxybenzeneacetic acid, DL-Dopa, L-Tyrosine	map00350
Latest HbA1c values	Citrate cycle	1.5898	0.05003	Cis-Aconitic acid	hsa00020
	Glycerolipid metabolism	1.1213	0.23676	Glycerol	hsa00561
	Vitamin B6 metabolism	0.67539	0.68759	Pyridoxal 5′-phosphate, Pyridoxal, 4-Pyridoxic acid	hsa00750
	Linoleic acid metabolism	0.08917	1.0	Linoleic acid, Glycerophosphocholine	hsa00591
	Caffeine metabolism	0.37079	0.69231	Paraxanthine, Caffeine	hsa00232
	Phenylalanine, tyrosine, and tryptophan biosynthesis	0.19682	1	L-Phenylalanine, L-Tyrosine,	hsa00400

## Data Availability

Data will be available upon request from the corresponding authors.

## Supplementary Materials

The following supporting information can be downloaded at: https://www.mdpi.com/article/10.3390/biom12070962/s1, Figure S1: Heatmaps of the complete list of metabolites.
