# Supplementary figures and images for: Untargeted Metabolomic Plasma Profiling of Emirati Dialysis Patients with Diabetes versus Non-Diabetic: A Pilot Study
Source: Biomolecules. 2022 Jul 8;12(7):962. doi: 10.3390/biom12070962 (PMC9313445; doi:10.3390/biom12070962)

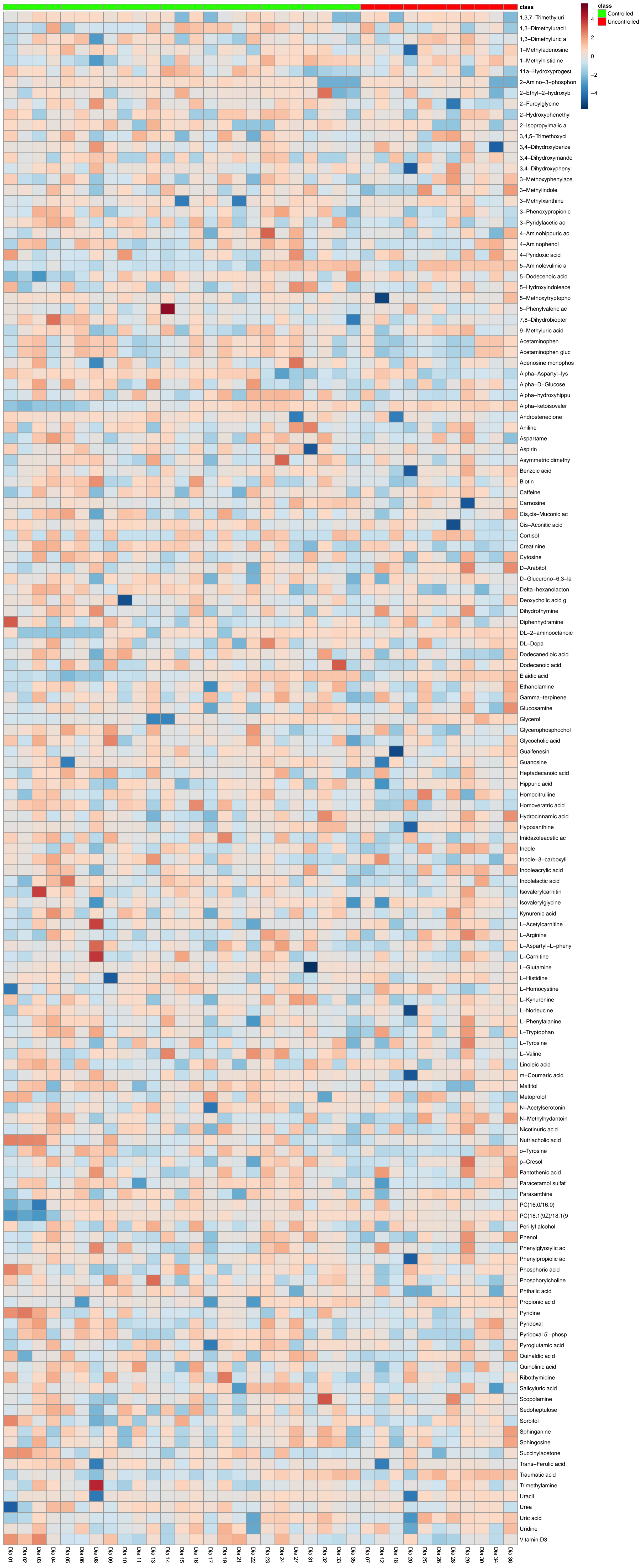

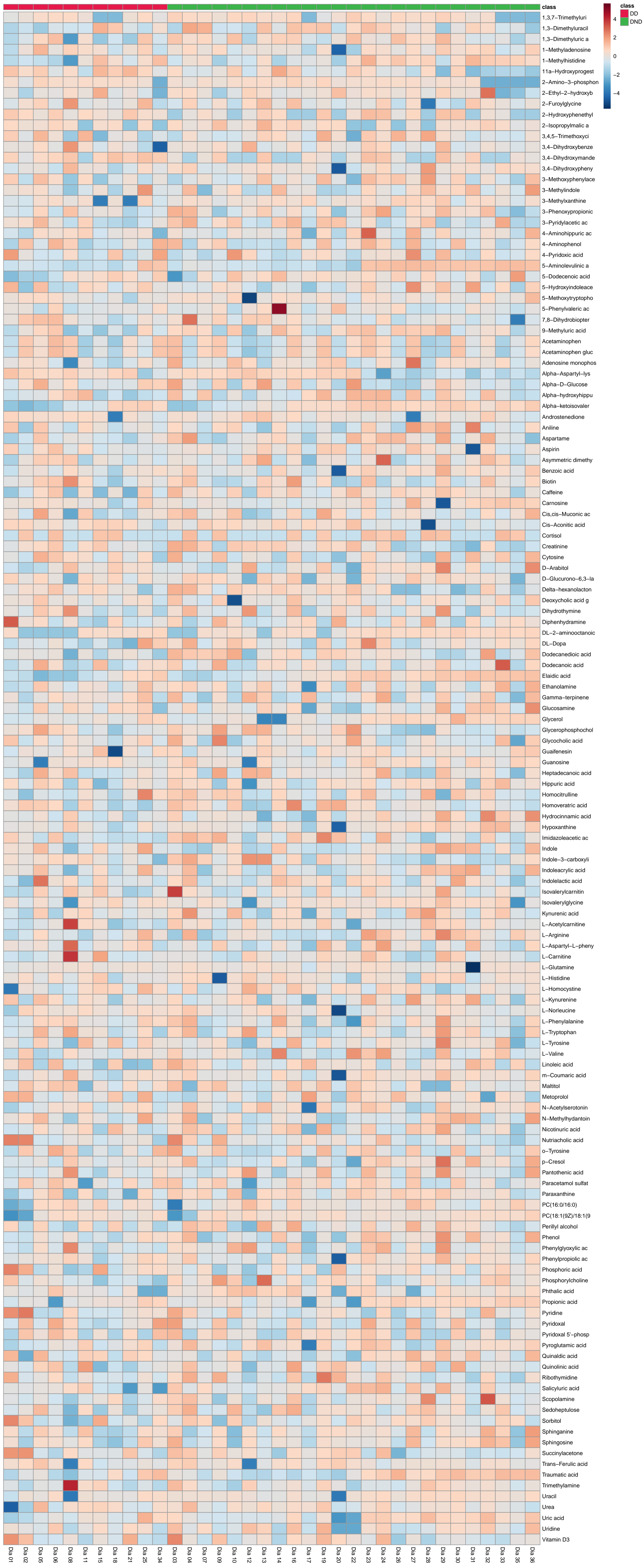

Supplement: Supplementary file 1 [file biomolecules-12-00962-s001.zip › Figure S1.pdf]
